# On the determinants of anti-COVID restriction and anti-vaccine movements: the case of *IoApro* in Italy

**DOI:** 10.1038/s41598-023-42133-x

**Published:** 2023-10-05

**Authors:** Vincenzo Alfano, Salvatore Capasso, Michele Limosani

**Affiliations:** 1 DiSEGIM, University of Napoli Parthenope, Naples, Italy; 2 Center for Economic Studies - CES-Ifo, Munich, Germany; 3 Department of Human and Social Sciences, Italian National Research Council, Rome, Italy; 4 University of Napoli Parthenope, Naples, Italy; 5 CSEF, University of Naples Federico II, Naples, Italy; 6 Department of Economics, University of Messina, Messina, Italy

**Keywords:** Health care, Public health, Quality of life

## Abstract

Following restrictions to control the spread of COVID-19, and subsequent vaccination campaigns, sentiments against such policies were quick to arise. While individual-level determinants that led to such attitudes have drawn much attention, there are also reasons to believe that the macro context in which these movements arose may contribute to their evolution. In this study, exploiting data on business activities which supported a major Italian anti-restriction and anti-vaccine movement, *IoApro*, using quantitative analysis that employs both a fractional response probit and logit model and a beta regression model, we investigate the relationship between socio-economic characteristics, institutional quality, and the flourishing of this movement. Our results suggest a U-shaped relationship between income and the proliferation of the movement, meaning that support for these movements increases the greater the degree of economic decline. Our results further indicate that the share of the population between 40 and 60 years old is positively related to support for such movements, as is institutional corruption.

## Introduction

Since the beginning of 2020, the world has been plagued by the COVID-19 virus. As a consequence, at the height of the pandemic a number of policies were enforced by governments all over the world, aiming to reduce the spread of the pandemic and forcing the vast majority of its inhabitants to significantly change their habits, to different extents. Such policies are known as non-pharmaceutical interventions (NPIs), and are aimed at enforcing social distancing and thereby limiting contagion. By the end of 2020 the best hope for people to return to their previous lifestyle, according to the majority of expert opinion, seemed to be a widespread vaccination campaign. By increasing personal resilience to the infection, hence reducing reproduction of the virus, and, more importantly, reducing its possibility of mutating into dangerous new forms, vaccines appeared to be the best way to fight COVID-19.

Several governments, especially among Western democracies, therefore put in place massive vaccination campaigns that prioritized various targets, seeking the best trade-off between safeguarding the frailer part of the population and allowing the productive part of society to come back to work. This seemed especially important to avoid further damaging an economic system that was already severely tried by several months of stay-at-home orders, lockdowns, and various restrictions that impeded trade. As a consequence, participatory community engagement soon emerged as an essential piece of the puzzle, to finally achieve widespread COVID-19 vaccination coverage among the public^1^, and hence come back to a normalized situation. In a dynamic and rapidly changing scenario, governments were trying to strike the right balance between restoring personal liberties, safeguarding public health via imposing restrictions, and reaching a level of immunity in the population that allowed coexistence with the virus. According to Burgess and her coauthors^1^, the same people who suffered disproportionate economic and health consequences from COVID-19 were asked to trust once again the very same institutions that had failed to provide adequate protection during the pandemic. Friction was naturally created, ensuring the perfect environment in which various anti-restriction and anti-vaccine movements could prosper, together with the proliferation of fake news about the effects of vaccines, and widespread levels of skepticism of different kinds.

It is notable that, while vaccines are undoubtedly very widely used and effective medical tools for disease prevention and hence boosting life expectancy, in recent years several controversies have arisen in this regard^2^. People’s concern about their safety post-vaccination has risen, and many uncontrolled reports of difficult provability have spread: from vaccines causing autism, to polio vaccine contamination with simian virus 40. As well as through coordinated efforts^3^, this is likely to have contributed to the spread of hysteria surrounding vaccine-associated risks, and resulted in a declining number of vaccinations in developed countries as well as several outbreaks of vaccine-preventable diseases (a famous example is measles) that have occurred in the heart of the developed Western world, such as in Europe and in North America, leading to some fatalities. The literature suggests that mistrust in vaccines is widespread in industrialized countries, with shares of skeptics well over the global average of 12%: according to Rosiello and coauthors^4^, the corresponding share is 41% in France, 21% in Italy, and 13% in the USA.

As could be imagined, possibly because of the very reasons highlighted by^1^, a similar skepticism and rhetoric were also in place in the case of the COVID-19 vaccine^5^. Indeed, after an initial period of enthusiasm, possibly driven by the promise of returning to normality thanks to the vaccine, the various vaccination campaigns raised many questions and widespread skepticism in several countries. As described in the previous case, in regard to the COVID-19 vaccine too this dynamic was especially present in democratic and more highly developed countries, which were also the first to have the vaccine available, and hence the first capable of organizing large-scale vaccination campaigns, which began as early as 2021. Shortly afterward there emerged protest movements against the vaccination campaign, also targeting the various policies designed to incentivize vaccination, such as the creation of vaccine passports to keep track of people’s vaccination status^6^, or restricted access for unvaccinated people to certain facilities (such as gyms or restaurants). It has been noted that a wave of discontent within the European Union (and more in general Western countries) was already in place before 2020, and that the pandemic crisis only exacerbated it^7,8^. As suggested by Dijkstra and his coauthors: “This discontent is purportedly driven by the very factors behind the surge of populism: differences in age, wealth, education, or economic and demographic trajectories”^9^. But with the pandemic crisis, and the consequent restrictions to business and everyday life, citizens began protesting in several parts of the world. While protests against COVID-19 restrictions occurred in all European countries, and generally throughout the Western world, their intensity varied greatly^10^. Some of these movements exhibited strategic behavior, such as in Germany, where several protest events were organized when COVID-19 mortality rates were low^10^; meanwhile, other protests were less strategic and quite violent. At times, the protest erupted in demonstrations against the government, such as in France, where over 100,000 people were reported as marching against the introduction of a vaccine passport (https://www.aljazeera.com/news/2022/1/9/more-than-100000-rally-in-france-against-covid-vaccine-rules, accessed on 21 April 2022), Canada, where the center of the capital city Ottawa was practically inaccessible for over a week (https://www.theguardian.com/world/2022/feb/07/ottawa-protests-explainer-vaccine-mandates-whats-next, accessed on 21 April 2022), New Zealand, where the police dismantled an encampment set up outside parliament (https://www.nbcnews.com/news/world/new-zealand-police-move-protest-covid-vaccine-mandate-rcna18240, accessed on 21 April 2022), and Italy, which we consider to be the most interesting case, and which is the focus of this article.

This study aims to provide, by means of quantitative analysis with the use of fractional probit and logit response and beta regression models, some evidence for the principal socio-economic and institutional characteristics related to the rise and flourishing of such movements in Italy. We consider it important to study the factors conducive to the rise of such movements: there are reasons to believe that even in 2023 the pandemic remains an important threat, due to its resurgence in some areas. Therefore, understanding the characteristics that led to the emergence of such movements may help policy-makers to better design future policies by gaining insight into their consequences. Moreover, these results may also be useful in the future, since epidemics and pandemics have been predicted to be an increasingly major threat in the years to come^11–13^. Future NPIs, vaccination campaigns, and health policies may therefore be fine-tuned. Furthermore, it seems important to highlight this link, since companies are closely linked to society as a whole, and the ethics of firms and society influence each other reciprocally^14^.

The rest of the paper is structured as follows: after this introduction, we present the Italian case in the second section, and in section three introduce the empirical strategy and present the data used in the analysis; the results are shown and discussed in section four, while conclusions are drawn in the last section.

## The Italian case: evolution of restrictions and the rise of the *IoApro* movement

We consider the Italian case particularly interesting with a view to studying the anti-restriction and anti-vaccination movements, and the socio-economic and institutional conditions that favored them. Importantly, Italy was the first Western country to be severely hit by the pandemic, for which it paid a very high price in terms of deaths^15–17^. According to the World Health Organization (WHO), in May 2023 the total number of COVID-19 cases in Italy had reached about 26 million confirmed cases, and over 190,000 deaths. Therefore, it seems reasonable to expect less skepticism toward vaccination and other policies designed to reduce the risk of dying from COVID-19. However, recent history and anecdotal evidence have proved that such expectations have been overstated.

With the outbreak of COVID-19 in late February 2020, the Italian government soon realized that the situation was getting out of control, with a frighteningly rapid multiplication of cases, especially in the north of the country. As a consequence, a harsh lockdown was imposed, which included mandatory closure of all businesses considered unessential, from March to May 2020^18^. Then, with a progressive reduction in new cases, thanks in part to a strict lockdown^18^, the government gradually lifted these restrictions during the summer of 2020. Chintalapuli et al.^19^ predicted that if the lockdown measure could have continued in Italy for another 60 days, the country would have experienced 35% fewer registered cases and 66% more recovered people. It was favored into lifting some restriction by a reduction of cases, but possibly also pressured into doing so by the protests of several stakeholders, such as business owners (especially in the tourism and leisure sector, a category for whom “losing” a summer was a crucial factor: https://www.ilfattoquotidiano.it/2020/08/12/coronavirus-scontro-governo-regioni-sulle-discoteche-maggiori-controlli-o-chiusure-ma-meta-dei-presidenti-si-oppongono/5898432/ accessed on 14/8/2023), unemployed (https://napoli.fanpage.it/25-aprile-a-napoli-protesta-dei-disoccupati-a-piazza-municipio-reddito-per-tutti/ accessed on 14/8/2023), political antagonist (https://www.romatoday.it/cronaca/manifestazione-roma-cosa-e-successo-oggi-30-maggio-2020.html, accessed on 14/8/2023), and activist (https://www.milanotoday.it/attualita/coronavirus/20-giugno-salviamo-lombardia.html accessed on 14/8/2023) since the spring and through the summer at the end of the first COVID-19 wave in 2020. At the time, the overall perception among the Italian population was generally that the emergency was over.

But with the arrival of autumn 2020, the number of cases began to rise again, and the government re-imposed a second, lighter and less generalized round of NPIs (which focused on school closures rather than business closures^20,21^). In this climate, with the arrival of 2021 a new movement emerged, that asked the government to reduce social distancing restrictions and allow many businesses that were shut down to halt the spread of the infection to re-open. It was called *IoApro*, which in Italian means “I open”, a clear reference to the willingness of business owners to re-open their activities and not be subject to further restrictions.

While initially *IoApro* was opposed solely to NPIs forcing business closures to prevent the spread of the contagion, with its increasing popularity it broadened its objectives. Especially with the peaks of the second and third COVID-19 waves in 2021, the movement began shifting its focus first toward an anti-restriction approach *tout court*, and then also to an anti-vaccine policy. Indeed, in order to boost the vaccination rate^22^, the new Italian government, led by Mario Draghi from March 2021, aiming to jump-start the vaccination campaign adopted from April 2021 onward a system of so-called vaccine passports. Its objective was to connect vaccination status with the lifting of restrictions, to incentivize citizens’ vaccination. As highlighted by the literature, pharmaceutical interventions (PIs) are, together with NPIs^15^, an essential part of the fight against the virus. The two kinds of policies are complementary, and their mix is an essential tool of any public health strategy. The basic idea of the Italian government’s strategy in 2021 was to differentiate the public according to their susceptibility to COVID, and therefore to let only those with the most protection (i.e. the vaccinated and those who had already had COVID-19 and therefore acquired natural immunity) to travel without restrictions outside their Italian region of residence and, from August 2021, to be allowed into several kinds of businesses, such as gyms and restaurants. The idea was also due to the widely held belief, even though the most recent literature has provided mixed evidence on this^23–26^, that those who were vaccinated or naturally immunized had a lower viral load, and were therefore less infectious and hence spread the virus less. Vaccine passports thus represented the link between PIs and NPIs.

The announcement and implementation of this strategy met with considerable resistance to the new policies, with some (which in the Italian case included conspiracy theorists in prominent and well-respected positions, for instance lawyers, doctors, and politicians) claiming that the strategy was scientifically unfounded and anti-constitutional. Very soon, widespread support among the public, and especially among small business owners, for the lockdown, which was seen as the quickest way to return to normal life, shifted to its opposite. This has been analyzed elsewhere with regard to the restaurant “Twittersphere”^27^. More generally, in the tense atmosphere of the time, many people, opposed to vaccination for various reasons, claimed they were being unfairly discriminated against, and dramatically compared the public health strategy enforced by the government to Nazi racial policies and South African apartheid. In this climate, the *IoApro* movement became increasingly popular (https://corrieredibologna.corriere.it/bologna/cronaca/21_ottobre_11/biagio-passaro-notorieta-ad-aprile-il-grido-ioapro-poi-parabola-ec98acb8-2a66-11ec-a8a7-97c00bb2b6f7.shtml accessed on 14/8/2023), possibly thanks to its widespread online presence, with the movement posting a series of videos on several social media sites protesting government regulations that forced businesses that did not comply with the new policies to close or be fined. The movement also gained some popularity by maintaining a lively presence on national television, where several of its leaders were invited to be interviewed on talk shows. This became a major issue in Italian public debate at the time, with strong media sensationalization and politicization of the topic^28^. The attitude was also referred to as pandemic denial^29^.

Given the imposition on many Italian business managers to check vaccine passports before granting access to their customers, a burden considered too great by many small business owners, and possibly also thanks to the general perception that another general lockdown like the one Italy had experienced in the spring of 2020 was not imminent, the focus of *IoApro* rapidly broadened, and shifted from being against NPIs to being against vaccine passports too and, in its more extremist fringe, against vaccines *tout court*. The movement exploited modern technologies and rapidly spread across the internet, advertising to like-minded people the presence of businesses, especially gyms, restaurants, and bars, that did not require their customers to be vaccinated in order to gain access. During the summer of 2021 those without vaccine passports were banned from entering many businesses, especially bars and restaurants, complicating life for the unvaccinated. As explained above, this was to achieve one of the objectives of the government’s strategy, namely to nudge people toward vaccination and increase its take-up rate.

*IoApro* adherents and followers began to openly challenge such policies, advertising the fact that they were opening regardless of any restrictions, and also, unlike what the laws prescribed, not checking the vaccination status of their customers. In other words, the movement was claiming that its adherents were open to all the public, regardless of the possession of vaccine passports and therefore vaccination status. This publicity was (for obvious reasons) carried out underground especially, thanks in part to a number of Telegram groups. Such groups, for instance “No more dictatorship” (https://www.ansa.it/english/news/general_news/2021/09/28/telegram-blocks-anti-vax-channel-for-inciting-violence_346438ad-7787-4cef-a3f1-9e8b9d1344ad.html, accessed on 26 May 2023), were often also connected to the spread of fake news and, generally, to anti-vaccine propaganda. Italian prosecutors asked Telegram (successfully) to close over fifty of these groups, due to the incitement to violence that was being carried out within them (http://www.news.cn/english/2021-09/07/c_1310173950.html, accessed on 26 May 2023).

Importantly for our analysis, this activity led at the very beginning of August 2021 to the creation of a personalized Google map, advertising over 300 businesses that were not, contrary to the law in force at the time, checking the vaccine passports of their customers before letting them in (https://www.open.online/2021/08/06/covid-19-green-pass-mappa-locali-io-apro/ (accessed on 21 April 2022). In other words, this tool (the map was available at https://www.google.com/maps/d/embed?mid=1INdrxWa1LXGRTBcb2oH_RioBGaS-ITIW up to at least November 2021, after which Google removed it due to violations of its terms. On 26 May 2023, it remains accessible through the Internet archive of *Wayback Machine*, at the URL: https://web.archive.org/web/20211105231206/, https://www.google.com/maps/d/embed?mid=1INdrxWa1LXGRTBcb2oH_RioBGaS-ITIW&ll=0%2C0&z=5) reported the list of businesses that were managed by people belonging, or in some way sympathetic, to the *IoApro* movement, and thus generally opposed to COVID-related restrictions and vaccine passports. While it is hard to generalize when talking about the anti-restriction and anti-vaccination movements, which comprise people from very different backgrounds, with very different aims, as well as different motivations underlying their distrust^30^, a considerable degree of overlap between these two movements may be assumed, especially in the context of a government strategy that relied on a mixture of PIs and NPIs, implemented jointly in order so that the latter would aid enforcement of the former.

## Methodology and data

### Research questions and empirical strategy

While previous contributions have focused on the determinants of vaccine hesitancy, both at a macro-level^31^, and often on a more individual level^32^, as is well presented in a recent systematic review^33^, surprisingly, there remains an important gap in the literature: investigation of the socio-economic characteristics that favor the emergence of anti-restriction and anti-vaccination movements is, to the best of our knowledge, still broadly overlooked. Research has focused on the determinants of NPI effectiveness^34–41^, but not, to the best of our knowledge, on the determinants that cause the rise of anti-restriction movements or sentiment. In this study, unlike previous contributions which, especially in the case of vaccine hesitancy, focused mostly on individual-level decisions^42–47^, we aim to shed light on the socio-economic and institutional quality characteristics, e.g. the macro conditions, that constitute an environment in which anti-restriction and anti-vaccine movements can flourish, in line with recent works that deal with other topics, such as energy policies^48^.

We believe that this approach can be helpful for the policy-maker to choose the optimal policy mix. While a good combination of PIs and NPIs may offer a way out of a pandemic^15^, overlapping of the two may also favor the spread of protest and organized movements. In this perspective, individual motivations, and studies relying on surveys and other micro data, cannot shed light on the habitat in which such movements flourish. Indeed, while individual-level data are for obvious reasons harder to consider and take into account when designing the optimal mix of policies, some precautions in the approach based on socio-economic data and characteristics of local institutions may be more easily implemented in the unfolding of a public health campaign. Finally, it is also important to point out that empirical research in this field is typically based on survey data, which for such sensitive topics are potentially biased by hesitancy to tell the truth in interviews. In short, an approach based on a macro framework may therefore be an interesting alternative and a complementary approach to the main one, overcoming some of its limits, and offering better and different insights into the mechanisms that trigger a good habitat for the development of such movements, integrating this strand of the literature.

In this regard, we believe that the Italian setting is a very useful case study that sheds some light on this relationship, for a number of reasons. First, Italy was the first Western country to be severely hit by COVID-19 and is therefore a good case study since the disease was widespread and was felt very much by the whole population. Second, Italian regions are very heterogeneous, both in terms of gross domestic product (GDP)^49^, social capital in its population^50–55^, and institutional quality^56–58^.

Hence, it seems to be a very good framework for a quantitative study, gathering data in the context of a homogeneous set of laws, policies, and government, and enabling us to study the impact of different socio-economic characteristics, which vary greatly country-wide. We aim to use this setting to test a number of hypotheses.

First, it may be assumed that people belonging to different generations may exhibit different attitudes toward the anti-vaccine movement^5^. This may be due to different cultures^44^, political ideology^59^, and education^60^. Such effects are certainly complex and non-linear, as has been proved by previous findings. For instance, as well as among those who are less educated, skepticism about vaccination is very present among those with the highest level of education^61,62^. While many of these characteristics make sense in individual-level studies, this is not so much the case in more aggregate analysis due to the variance in the statistical unit. Moreover, the small number of Italian provinces would not allow us to have enough observations to include all such variables. At the same time, we believe that these characteristics are much more similar and constant in people who belong to the same age band.

Furthermore, the literature has so far presented mixed evidence on whether age impacts support for anti-COVID restrictions movements. Some research suggests that younger people are more likely to oppose restrictions: Spaccatini and her coauthors^63^ found that younger people’s ageism towards older adults predicted support for isolating older people and selective lockdowns of older populations, and Gerace et al.^64^ found that younger age predicted opposition to easing COVID restrictions in the U.S. However, there are other reasons to believe that age was not a strong predictor. The same work^64^ also found that political affiliation, social conservatism, and experiences with COVID-19 were stronger predictors of attitudes than age alone. The relationship between age and support for anti-restriction movements may hence depend on other determinants, such as the political context.

In summary, while some research points to younger people opposing COVID restrictions more than older people, the evidence is mixed and may depend on political and social context. More research is needed to fully understand how age impacts views on COVID-19 policy, and we aim to contribute to efforts to solve this puzzle by testing the role of age in our context. Hence, it seems worthwhile to investigate this discontinuity, which leads us to stipulate RQ1:

- RQ1: Which age bands in the population increase the spread of these movements?

There are reasons to believe that socio-economic status, as suggested by a strand of this literature, is positively correlated with pro-vaccination behavior^5^. Indeed, the findings suggest that the lower one’s socio-economic status, the higher the tendency to be opposed to vaccination^42,43^. Moreover, as highlighted by previous literature^47^, it may be assumed that getting vaccinated can depend on an individual’s perception of the costs and benefits linked to this choice. Finally, it is important to highlight that places that suffered economic decline and, following a seminal definition by Rodríguez-Pose, the “places that don’t matter”^7^, exhibit support for an anti-systemic stance (and electoral support for anti-system political parties). Consensus regarding anti-restrictions and anti-vaccination movements could potentially follow a similar dynamic, given their anti-government nature. Hence, it seems that the support for this movement may also be driven by economic factors^8^. This leads us to RQ2.1:

- RQ2.1: What is the relationship between average income and the spread of these movements?

Other than absolute levels of income, previous works have suggested that populist stances did not take hold in the poorest areas of a country, but found fertile terrain in areas that had suffered economic decline^7^. In other words, it is not the level of welfare that directly triggers this support, but the loss of it. Once again, support for these movements can probably, at least to some extent, be considered as following similar dynamics to the anti-restriction movements we are studying, due to common roots in anti-government movements and populism. It thus seems worthwhile to test the hypothesis that economic decline, rather than absolute level of wealth, leads to a rise in support for anti-restrictions movements. This seems an especially interesting assumption since COVID-19 has undoubtedly been an economic cost for a large part of the population, and hence, as stated above, that may be an important trigger for the support for such stances. We test this hypothesis in RQ 2.2:

- RQ2.2: Does economic decline have an impact on the spread of these movements?

Moreover, as is often the case when dealing with income and socio-economic status, this relationship may be more complex than a simple linear one. It may exhibit diminishing marginal returns, and be variably influenced by the perception of belonging to a different level of welfare in a society. Hence, it is possible that this relationship could be non-linear—more precisely, quadratic—and gain the support (as has already been suggested for education level in the case of vaccination^61,62^) of the poorest and richest parts of society. We test this hypothesis in RQ 2.3:

- RQ2.3: Is there a quadratic relationship between average income and the spread of these movements?

While vaccination is often treated in the relevant empirical literature, due to obvious limits intrinsic to quantitative analysis, as a homogeneous phenomenon, there are reasons to believe that the COVID-19 pandemic had its own characteristics that set it apart from other diseases: COVID-19 was the first pandemic to take place in an information-intensive society, and the other diseases for which a vaccine is available are now both less widespread and better known, both among the public and among health practitioners. Therefore, it is worth exploring whether the unfolding of the pandemic had any impact on the spread of anti-COVID restrictions and vaccination movements. This leads to RQ3:

- RQ3: What is the relationship between the unfolding of the COVID-19 pandemic and the spread of these movements?

Finally, one might suppose that the institutional quality of the geographical areas in question may play a role in this relationship. Corruption, government effectiveness, regulatory quality, rule of law, and voice and accountability are all dimensions of the effectiveness of local institutions, which can easily affect the way people view the anti-restriction and anti-vaccine movements, influencing trust in institutions and hence the behavior of citizens. Therefore, we may stipulate RQ4 as follows:

- RQ4: What is the relationship between institutional quality and the spread of these movements?

### Method and data

To answer our research questions, using quantitative analysis applied to Italian data concerning the presence of businesses listed in the *IoApro* map by province, we modeled the presence of the movement with Eq. (1):

1 $${AntiVax}_{p}=\alpha +{\beta }_{1}{SocioEco}_{p}+{\beta }_{2}{COVID}_{p}+{\beta }_{3}{IQI}_{p}+\varepsilon ,$$

where our operationalization of the spread of the anti-vaccine movement $AntiVax$ is the per capita total number of *IoApro* businesses listed in the Google map described above (population as of 1 January 2021, gathered from the Italian National Statistics Agency, ISTAT). Data were gathered on 26 August 2021 from the website https://www.google.com/maps/d/embed?mid=1IndrxWa1LXGRTBcb2oH_RioBGaS-ITIW. This variable is modeled as a function of:

- $SocioEco$, a matrix of seven variables describing the socio-economic characteristics of the population in province *p*. The first four variables are labeled *ShPop 20–30, ShPop 30–40, ShPop 40–50, ShPop 50–60*, and represent the share of the provincial population belonging to Generation Z, Millennials, Generation X and Baby Boomers: in other words, the share of local population aged between 20 and 30 years old, 30 and 40, and so on (data once again from ISTAT, as of 1 January 2021). In this way, we aim to test whether there is an age band which is more likely to adhere to such a movement (or at any rate increase its numbers), thereby answering *RQ1* (please note that not all the age bands are included, to avoid obvious problems of multicollinearity in the estimates). Moreover, the matrix also includes *Income* and *Income sq*., two variables with the average taxable income per capita, expressed in thousands of euro, for each province *p* (data from the Italian Revenue Agency, referring to 2019), to check for the linear and quadratic effects of average wealth on the proliferation of the anti-vaccine movement, and *Rel.Inc.Dec.*, a variable expressing which decile of the distribution of the relative 2020 average income, with respect to the one of 2019, the province belongs to. In other words, this variable measures the relative economic decline in 2020, with respect to 2019, of each Italian province *p*. It is important to highlight that the use of deciles instead of a ratio allows us to avoid issues concerning both multicollinearity and correlation between independent variables. All these variables allow us to answer *RQ2.1, RQ 2.2*, and *RQ 2.3*;
- $COVID$, a matrix of three variables describing the impact of COVID-19 in the province over the course of 2020, to answer *RQ3*. It comprises the variables *Sh.Under30k, PopDens*, and *Tot.Cases ph*. The first of these is the share of the province’s population that lives in municipalities with fewer than 30,000 inhabitants. This, following previous contributions^20^, is an operationalization that lets us control for the structural characteristics of the province *p*. In other words, by measuring how many people in the province are living in small population centers (and conversely how many live in more urban areas) we control for the presence of social scarring and other distinguishing features that may affect the propensity to support the *IoApro* movement, as well as for the necessity to commute towards bigger centers for daily needs, which means being more severely affected by COVID-19 restrictions. The second variable is population density, i.e. the number of inhabitants per square kilometer of the province, under the assumption that the higher the density, the greater the perceived threat of the pandemic. Finally, the third variable is the total number of COVID-19 cases registered in the province on 31/7/2021, and hence a measure of the severity with which the COVID-19 pandemic hit province *p*;
- $IQI$, a matrix of five variables describing the institutional quality of province *p*, in order to answer *RQ4*. It consists of the different scores of the Institutional Quality Index (IQI) present in the literature^56^, a composite index inspired by the World Governance Indicator^65^, in its latest update (data referring to 2019). It is worth noting that the IQI has five different scores^56^: corruption, government effectiveness, regulatory quality, rule of law, and voice and accountability. All details are well addressed on the IQI website and publications: in our case it is important to highlight that it captures at a provincial level the quality of public service and the policies formulated and implemented by the local government, as well as individuals’ perception of law enforcement in terms of contract fulfillment, property rights, and policing. Unfortunately, the Sud Sardegna province, suppressed in 2021, was not included in the last update of IQI. As a consequence, regressions including this matrix have one observation less.

**Figure 1:**
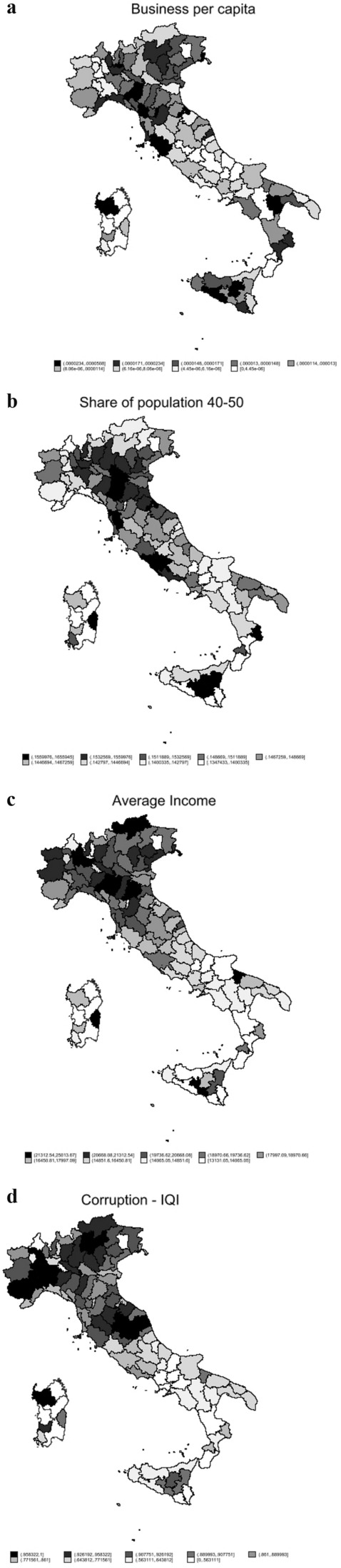
(**a**) Heat map of business per capita. (**b**) Heat map of share of generation X. (**c**) Heat map of average income. (**d**) Heat map of corruption IQI.

Gathering the above data led to the creation of a dataset consisting of 107 observations, one for each Italian province. Figure 1a to d show a heat map of the most important variables for the Italian provinces, while Table 1 presents descriptive statistics for all the variables in the dataset.

**Table 1:** Descriptive statistics.

Label	Variable	Obs	Mean	Std. dev	Min	Max
Businesses pc	Number of business reported on the *IoApro* map for each province *p*, divided by the population of the province itself	107	0.0000133	9.35e-06	0	0.0000508
ShPop 20–30	Share of population of province *p* aged between 20 and 30 years old	107	0.1007756	0.0099541	0.0831864	0.1244787
ShPop 30–40	Share of population of province *p* aged between 30 and 40 years old	107	0.113	0.0081551	0.0917963	0.1307099
ShPop 40–50	Share of population of province *p* aged between 40 and 50 years old	107	0.1480125	0.0062437	0.1347433	0.1655945
ShPop 50–60	Share of population of province *p* aged between 50 and 60 years old	107	0.1584012	0.0055833	0.1443081	0.1703471
Income	Total tax revenues on salary for each province *p* divided by the population of the relative province, in thousands of euro	107	1.80529	.2997923	1.313105	2.501367
Income sqr	Total tax revenues on salary for each province *p* divided by the population of the relative province, in thousands of euro, squared	107	3.348106	1.086041	1.724246	6.256838
Rel.Inc.Dec	Decile for the relative income variable. This is built as the ratio between the total tax revenues on salary for each province *p* in 2020 and in 2019	107	5.457944	2.885489	1	10
Sh.Under30k	Share of the population in the province *p* that lives in municipalities under 30,000 inhabitants	107	0.6245704	0.194124	0.1287831	1
PopDens	Population of province *p* divided by the respective area in square kilometers	107	270.1714	384.5909	35.74888	2632.711
Tot cases ph	Total COVID-19 cases reported in the province on 31/7/2021	107	6763.298	2039.23	2786.703	13,869.53
Corr-IQI	Score for province *p* in 2019 of the corruption index as per the Institutional Quality Index (Nifo and Vecchione^56^)	106	0.7922553	0.1778886	0	1
Gov-IQI	Score for province *p* in 2019 of the government effectiveness index as per the Institutional Quality Index (Nifo and Vecchione^56^)	106	0.4051604	0.190254	0	1
RQ-IQI	Score for province *p* in 2019 of the regulatory quality index as per the Institutional Quality Index (Nifo and Vecchione^56^)	106	0.475188	0.2125494	0	1
RoL-IQI	Score for province *p* in 2019 of the rule of law index as per the Institutional Quality Index (Nifo and Vecchione^56^)	106	0.569323	0.242771	0	1
Voice-IQI	Score for province *p* in 2019 of the voice and accountability index as per the Institutional Quality Index (Nifo and Vecchione^56^)	106	0.5350013	0.1794268	0	1

## Results

Equation (1) has as its dependent variable a rate, i.e. the number of anti-vax-friendly businesses in the province divided by population. Therefore, the natural candidate for the above estimation would appear to be a fractional response model^66–68^. Actually, as highlighted by the relevant literature, fractional regression is a well-developed alternative for modelling bounded dependent variables. In principle, these models are similar to ordered logit regression, but are more flexible in that the dependent variable can be measured as continuous over a defined bounded range.

The results of the estimation of (1) using a probit model for the conditional mean, with robust standard errors^69–71^, are shown in Table 2. The different matrixes in (1) were added individually in the regression to control for possible collinearity issues. As can be observed, in all the different model specifications (2.1, 2.2, and 2.3) the share of the population between 40 and 50 years old is statistically significantly related to the presence of anti-vax businesses, as is both the average income and its square, other than the decile of relative income. This suggests that such variables are correlated to the flourishing of anti-vax movements. To be precise, the model suggests that an increase in the share of the province population in the age band 40–50 is correlated to an increase in the spread of such businesses in the province. This partially answers RQ1, regarding the generation that is most likely to support such movements (so-called Generation X). Furthermore, the share of the population in the age band between 50 and 60 is also statistically significant in the post-complete specification (2.3). While 10% is not a particularly significant threshold, note that the regression includes only 106 observations, and therefore such a result is not trivial.

**Table 2:** Fractional response model—Probit.

	(2.1)	(2.2)	(2.3)
	Businesses pc	Businesses pc	Businesses pc
ShPop 20–30	− 2.271	− 0.814	1.178
	(− 0.74)	(− 0.26)	(0.35)
ShPop 30–40	− 5.098	− 5.194	− 3.467
	(− 1.37)	(− 1.41)	(− 0.83)
ShPop 40–50	6.957**	7.337**	5.941*
	(2.33)	(2.45)	(1.78)
ShPop 50–60	3.554	4.119	6.833*
	(1.02)	(1.11)	(1.67)
Income	−1.846***	− 2.006***	− 2.509***
	(− 2.67)	(− 2.72)	(− 2.82)
Income sqr	0.426**	0.493***	0.601***
	(2.44)	(2.65)	(2.66)
Rel.Inc.Dec	− 0.0204***	− 0.0229***	− 0.0185***
	(− 3.45)	(− 3.69)	(− 2.74)
Sh.Under30k		− 0.0279	− 0.0121
		(− 0.39)	(− 0.13)
PopDens		− 0.0000712*	− 0.0000232
		(− 1.84)	(− 0.38)
Tot.Cases ph		− 0.00000997	− 0.0000117
		(− 1.29)	(− 1.40)
Corr-IQI			0.482**
			(2.51)
Gov-IQI			0.0679
			(0.62)
RQ-IQI			0.121
			(1.32)
RoL-IQI			0.0277
			(0.23)
Voice-IQI			− 0.243
			(− 1.19)
Constant	− 2.981***	− 3.083***	− 3.549***
	(− 2.75)	(− 2.87)	(− 2.76)
Observations	107	107	106
Ps.R sqr	0.00400	0.00438	0.00591
Log-likelihood	− 0.0173	− 0.0173	− 0.0171
Akaike’s criterion	16.03	22.03	32.03
Schwarz’s Bayesian information	37.42	51.44	74.65

*t* statistics in parentheses.

*p < 0.1, **p < 0.05, ***p < 0.01.

**Figure 2:**
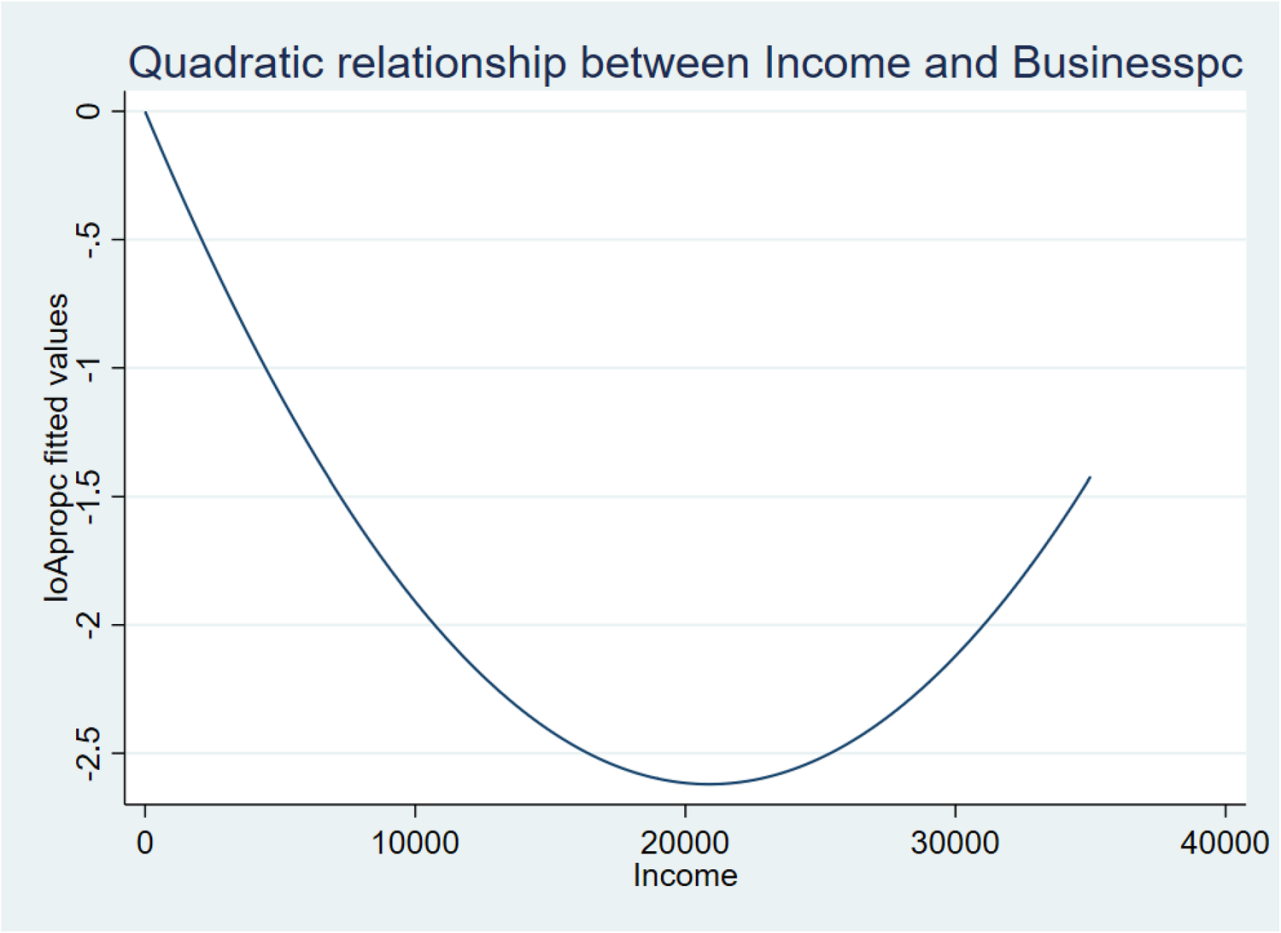
Quadratic impact of *Income* on *Businesspc*.

As regards income, the negative coefficient of the linear term and the positive of the quadratic suggest that a U-shaped relationship is in place. Hence, the higher the average income of the province, the lower the popularity of such movements, up to a certain threshold (which our most complete specification, 2.3, estimates at €20,901), after which the relationship becomes positive, and higher income corresponds to higher support. The turning point in value is €2,848.1 above the average value (€18,052.9 in the sample), suggesting that other than the poorest part of the society, in provinces considerably above the average (over a sixth above the average value) there is higher support for anti-restriction movements. The parabolic curve described by our most complete model (2.3) is represented in Fig. 2. Moreover, the negative coefficient of *Rel.Inc.Dec.* suggests that the greater the economic decline suffered by a province in 2020 (i.e. provinces belonging to the lower deciles of the 2020 income divided by the 2019 income), the higher the support for these movements in that province will be. This is in line with what has already been suggested by the literature^7^ regarding support of populist movements: economic decline spurs it, and it is a cost to be taken into account when computing the cost–benefit analysis of welfare policies.

The only variable in the COVID-related matrix to show some statistical significance, in either (2.2) or (2.3), is *PopDens* in (2.2). This is negative, suggesting that more densely populated provinces showed less support for *IoApro*, though this result does not seem particularly important, since it disappears when we control for institutional quality, indicating that this matters more. Overall, these results suggest that how the pandemic unfolds is not at all, or poorly, related to the sentiment for or against COVID restrictions and vaccination. Some anecdotal evidence already suggested this, as in the case of Italy when viewed from a cross-country perspective: this is a country that had important anti-restriction and anti-vaccination movements even though it was among the countries most severely hit by the pandemic.

Finally, as regards the different dimensions of institutional quality in (2.3), our findings suggest that these movements spread more in provinces that have higher levels of corruption. Hence, these findings suggest the importance of fighting corruption, also in this specific setting.

To check the robustness of these preliminary findings, we replicated the regression analysis in Table 2 using a fractional response logit model, instead of the probit. The results, presented in Table 3, are consistent with our main findings. Similar results are obtained once again when employing a beta regression estimator with robust standard errors. The findings are detailed in Table 4. It is worth noting that in this latter robustness test, five observations are lost as a consequence of having five provinces with *Businesspc* values equal to 0. Consequently, these observations must be excluded from the regression analysis due to the intrinsic requirement of the estimator, which necessitates a dependent variable strictly greater than 0 (and strictly lower than 1)^72,73^. Hence, it is possible to state that there is at least some evidence of robustness in our estimates, provided by equivalent results obtained by different estimators. Appendix 1 presents marginal effects of the estimations in Tables 2, 3, and 4.

**Table 3:** Fractional response model—Logit.

	(3.1)	(3.2)	(3.3)
	Businesses pc	Businesses pc	Businesses pc
ShPop 20–30	− 10.06	− 3.817	5.066
	(− 0.74)	(− 0.28)	(0.34)
ShPop 30–40	− 22.61	− 23.06	− 15.21
	(− 1.38)	(− 1.42)	(− 0.82)
ShPop 40–50	30.63**	32.32**	26.13*
	(2.34)	(2.46)	(1.78)
ShPop 50–60	16.06	18.35	30.74*
	(1.05)	(1.13)	(1.71)
Income	− 8.160***	− 8.916***	− 11.35***
	(− 2.69)	(− 2.74)	(− 2.83)
Income Sqr	1.882**	2.192***	2.729***
	(2.45)	(2.67)	(2.67)
Rel.Inc.Dec	− 0.0899***	− 0.101***	− 0.0815***
	(− 3.46)	(− 3.68)	(− 2.74)
Sh.Under30k		− 0.117	− 0.0628
		(− 0.37)	(− 0.15)
PopDens		− 0.000316*	− 0.000126
		(− 1.81)	(− 0.46)
Tot.Cases ph		− 0.0000436	− 0.0000519
		(− 1.29)	(− 1.42)
Corr-IQI			2.184**
			(2.54)
Gov-IQI			0.302
			(0.63)
RQ-IQI			0.538
			(1.33)
RoL-IQI			0.125
			(0.24)
Voice-IQI			− 1.083
			(− 1.18)
Constant	− 5.865	− 6.220	− 8.204
	(− 1.23)	(− 1.31)	(− 1.44)
Observations	107	107	106
Ps.R sqr	0.00403	0.00441	0.00597
Log-likelihood	− 0.0173	− 0.0173	− 0.0171
Akaike’s criterion	16.03	22.03	32.03
Schwarz’s Bayesian information	32.74	51.44	74.65

*t* statistics in parentheses.

*p < 0.1, **p < 0.05, ***p < 0.01.

**Table 4:** Beta regression model.

	(4.1)	(4.2)	(4.3)
	Businesses pc	Businesses pc	Businesses pc
ShPop 20–30	− 1.225	3.753	9.102
	(− 0.10)	(0.30)	(0.65)
ShPop 30–40	− 23.62*	− 24.66*	− 20.24
	(− 1.66)	(− 1.74)	(− 1.23)
ShPop 40–50	26.37**	28.49**	24.92**
	(2.23)	(2.40)	(1.98)
ShPop 50–60	18.82	19.23	28.30*
	(1.45)	(1.47)	(1.73)
Income	− 7.200***	− 8.047***	− 8.651**
	(− 2.80)	(− 2.96)	(− 2.29)
Income Sqr	1.664***	1.962***	2.099**
	(2.58)	(2.91)	(2.23)
Rel.Inc.Dec	− 0.0916***	− 0.102***	− 0.0836***
	(− 4.39)	(− 4.69)	(− 3.08)
Sh.Under30k		− 0.0201	0.148
		(− 0.08)	(0.41)
PopDens		− 0.000260**	− 0.000155
		(− 1.99)	(− 0.70)
Tot.Cases ph		− 0.0000271	− 0.0000253
		(− 1.00)	(− 0.78)
Corr-IQI			1.509*
			(1.90)
Gov-IQI			0.0969
			(0.20)
RQ-IQI			0.387
			(1.08)
RoL-IQI			− 0.165
			(− 0.36)
Voice-IQI			− 0.960
			(− 0.99)
Constant	− 7.392*	− 7.305*	− 9.689*
	(− 1.84)	(− 1.83)	(− 1.80)
Scale			
Constant	12.45***	12.48***	12.55***
	(84.53)	(85.66)	(95.82)
Observations	102	102	101
Log-likelihood	1073.4	1074.8	1067.7
Akaike’s criterion	− 2128.9	− 2125.7	− 2101.5
Schwarz’s Bayesian information	− 2105.2	− 2094.2	− 2057.0

*t* statistics in parentheses.

*p < 0.1, **p < 0.05, ***p < 0.01.

## Conclusions

After the outbreak of the COVID-19 pandemic in 2020, hopes for “a new normality” depended largely on the creation of herd immunity, and hence on higher levels of immunity in the population. In the context of a rapidly mutating virus that challenges the human ability to produce effective antibodies for a long time, the best strategy would appear to be a mix of NPIs, in order to reduce the *Rt* and therefore the number of virus variants, and a successful vaccination campaign, to foster immunity among citizens. Nonetheless, over the course of 2021 there emerged a considerable proportion of the public, perhaps wearied by a long lockdown, and more generally by pandemic fatigue, that was skeptical about the actual implementation of the above strategy. This was especially the case in the Western world. In our study, adopting an original macro-level approach, which, to the best of our knowledge, has not been used widely in the literature, and a novel operationalization of the spread of anti-restriction and anti-vaccine sentiment that relies on the spread of the *IoApro* business-friendly movement in Italy, we investigated four different research questions about the relationship between socio-economic and institutional quality characteristics and the spread of such sentiments.

Our analysis found evidence of a U-shaped relationship between the spread of such movements and provincial average income. In other words, while at low income levels an increase corresponds to a reduction in the spread of such movements, this relationship declines as average income grows until the sign is switched and the impact is thus reversed. At high income levels, the higher the average level, the more widespread anti-restriction and anti-vaccine movements are, possibly also because at higher incomes the likelihood of having a business is higher, and hence there are more firm owners who will potentially join the movement. Also, our findings suggest a role of economic decline in fostering support of the population for these movements. Indeed, provinces where the 2020 income is closer or even higher to that of 2019 exhibit lower levels of support for *IoApro*.

Moreover, provinces with higher shares of residents aged between 40 and 60 are more susceptible to such movements. This finding especially holds for so-called Generation X, the 40–50 year old age band. While there is no evidence regarding the role in this relationship of the severity with which the COVID-19 pandemic affected a province, that of institutional quality is evident: corruption is the most important area. Indeed, areas with higher levels of corruption exhibit a higher spread of these movements.

All this suggests that institutional quality and economic development matter, in this context as well. In this regard, an implicit consequence of our findings is that regional characteristics should be taken into due consideration by policymakers when implementing policies, since unexpected and detrimental effects can easily backfire, and crowd out original intentions. While it is beyond the scope of this exploratory article to provide a comprehensive theory that could link populism and anti-restriction and anti-vaccination movements, or to state whether the “places that don’t matter”, to quote a popular definition^7^, are those that protested the most against COVID-19 restrictions and vaccines, it is notable that none of the top 15 provinces supporting the *IoApro* movement is an Italian regional capital (*capoluogo di provincia*). These provinces exhibit an important degree of geographical disparity, and can be found in the north, the centre, the south, and the islands. It is hard to detect the red thread running between them, but it seems initially that none of these provinces are among those most prominent in economic or political terms in Italy.

To the best of our knowledge, this article brings the first empirical evidence of the relationship between the spread of an anti-restriction and anti-vaccine movement arising in Italy as a reaction to the implementation of PIs and NPIs. We believe that this is an interesting addition to the literature, which so far has focused on these movements only as a result of PIs and NPIs, in other words considering COVID-19 as a determinant, and not according to the socio-demographic characteristics of the territories. Nonetheless, our study does have its limitations, of which the reader should be warned. The Italian case offers a particularly interesting framework for the relationship in question, because of the reasons explained above. Nonetheless, given that our analysis focuses on Italy, and specifically on the *IoApro* movement, its external validity may be limited. Caution is suggested in applying our findings to other contexts. Second, the statistical power of the study is limited by the paucity of the sample, which nonetheless draws, so far as we are aware, on the best data available. To this respect, it should also be acknowledged that it is unclear how comprehensive the Google map of *IoApro*-sympathising businesses is. While we believe these data are interesting since they capture the willingness to belong to the movement, it is important to recognize that may not be exhaustive, and hence may not be entirely representative of each province. Even though a strong correlation between the real support towards *IoApro* and our operationalization would in any case justify our findings, it is important to recognize this major limitation. Third, our operationalization of the spread of the *IoApro* movement, while novel and interesting insofar as it measures a concept that is intrinsically difficult to measure, is not exempt from potential biases due to differences among territories.

For all the above reasons we believe that further studies are needed, focusing on other countries, in order to test the validity of these results in other contexts, or using different operationalizations of the spread of anti-restriction and anti-vaccine sentiments. Moreover, the implementation of spatial models could provide an interesting direction for studying potential spill-over and other effects.

## Data Availability

The data that support the findings of this study are available from the corresponding author, upon reasonable request.

## Appendix 1—Marginal effects

See Tables 5, 6 and 7.

**Table 5:** Marginal effects-fractional probit.

	(A1.1)	(A1.2)	(A1.3)
	Businesses pc	Businesses pc	Businesses pc
ShPop 20–30	− 2.271	− 0.814	1.178
	(− 0.74)	(− 0.26)	(0.35)
ShPop 30–40	− 5.098	− 5.194	− 3.467
	(− 1.37)	(− 1.41)	(− 0.83)
ShPop 40–50	6.957**	7.337**	5.941*
	(2.33)	(2.45)	(1.78)
ShPop 50–60	3.554	4.119	6.833*
	(1.02)	(1.11)	(1.67)
Income	−1.846***	− 2.006***	− 2.509***
	(− 2.67)	(− 2.72)	(− 2.82)
Income Sqr	0.426**	0.493***	0.601***
	(2.44)	(2.65)	(2.66)
Dec.of Rel.Inc	− 0.0204***	− 0.0229***	− 0.0185***
	(− 3.45)	(− 3.69)	(− 2.74)
Sh.Under30k		− 0.0279	− 0.0121
		(− 0.39)	(− 0.13)
PopDens		− 0.0000712*	− 0.0000232
		(− 1.84)	(− 0.38)
Tot.Cases ph		− 0.00000997	− 0.0000117
		(− 1.29)	(− 1.40)
Corr-IQI			0.482**
			(2.51)
Gov-IQI			0.0679
			(0.62)
RQ-IQI			0.121
			(1.32)
RoL-IQI			0.0277
			(0.23)
Voice-IQI			− 0.243
			(− 1.19)
Observations	107	107	106

Marginal effects, *t* statistics in parentheses.

(d) for discrete change of dummy variable from 0 to 1.

**p* < 0.1, ***p* < 0.05, ****p* < 0.01.

**Table 6:** Marginal effects-fractional logit.

	(A2.1)	(A2.2)	(A2.3)
	Businesses pc	Businesses pc	Businesses pc
ShPop 20–30	− 10.06	− 3.817	5.066
	(− 0.74)	(− 0.28)	(0.34)
ShPop 30–40	− 22.61	− 23.06	− 15.21
	(− 1.38)	(− 1.42)	(− 0.82)
ShPop 40–50	30.63**	32.32**	26.13*
	(2.34)	(2.46)	(1.78)
ShPop 50–60	16.06	18.35	30.74*
	(1.05)	(1.13)	(1.71)
Income	− 8.160***	8.916***	11.35***
	(− 2.69)	(− 2.74)	(− 2.83)
Income Sqr	1.882**	2.192***	2.729***
	(2.45)	(2.67)	(2.67)
Dec.of Rel.Inc	− 0.0899***	− 0.101***	− 0.0815***
	(− 3.46)	(− 3.68)	(− 2.74)
Sh.Under30k		− 0.117	− 0.0628
		(− 0.37)	(− 0.15)
PopDens		− 0.000316*	− 0.000126
		(− 1.81)	(− 0.46)
Tot.Cases ph 31/7		− 0.0000436	− 0.0000519
		(− 1.29)	(− 1.42)
Corr-IQI			2.184**
			(2.54)
Gov-IQI			0.302
			(0.63)
RQ-IQI			0.538
			(1.33)
RoL-IQI			0.125
			(0.24)
Voice-IQI			− 1.083
			(− 1.18)
Constant	− 5.865	− 6.220	− 8.204
	(− 1.23)	(− 1.31)	(− 1.44)
Observations	107	107	106

*t* statistics in parentheses.

**p* < 0.1, ***p* < 0.05, ****p* < 0.01.

**Table 7:** Marginal effects-beta regression.

	(A3.1)	(A3.2)	(A3.3)
	Businesses pc	Businesses pc	Businesses pc
ShPop 20–30	− 1.225	3.753	9.102
	(− 0.10)	(0.30)	(0.65)
ShPop 30–40	− 23.62*	− 24.66*	− 20.24
	(− 1.66)	(− 1.74)	(− 1.23)
ShPop 40–50	26.37**	28.49**	24.92**
	(2.23)	(2.40)	(1.98)
ShPop 50–60	18.82	19.23	28.30*
	(1.45)	(1.47)	(1.73)
Income	7.200***	8.047***	8.651**
	(− 2.80)	(− 2.96)	(− 2.29)
Income Sqr	1.664***	1.962***	2.099**
	(2.58)	(2.91)	(2.23)
Dec.of Rel.Inc	− 0.0916***	− 0.102***	− 0.0836***
	(− 4.39)	(− 4.69)	(− 3.08)
Sh.Under30k		− 0.0201	0.148
		(− 0.08)	(0.41)
PopDens		− 0.000260**	− 0.000155
		(− 1.99)	(− 0.70)
Tot.Cases ph 31/7		− 0.0000271	− 0.0000253
		(− 1.00)	(− 0.78)
Corr-IQI			1.509*
			(1.90)
Gov-IQI			0.0969
			(0.20)
RQ-IQI			0.387
			(1.08)
RoL-IQI			− 0.165
			(− 0.36)
Voice-IQI			− 0.960
			(− 0.99)
Observations	102	102	101

Marginal effects, *t* statistics in parentheses.

(d) for discrete change of dummy variable from 0 to 1.

**p* < 0.1, ***p* < 0.05, ****p* < 0.01.
